# Metformin Mitigates Sepsis-Related Neuroinflammation *via* Modulating Gut Microbiota and Metabolites

**DOI:** 10.3389/fimmu.2022.797312

**Published:** 2022-04-29

**Authors:** Huayan Zhao, Yuanjun Lyu, Ruiqing Zhai, Guiying Sun, Xianfei Ding

**Affiliations:** ^1^Department of Critical Care Medicine, The First Affiliated Hospital of Zhengzhou University, Zhengzhou, China; ^2^Department of Respiratory, The First Affiliated Hospital of Zhengzhou University, Zhengzhou, China; ^3^College of Bioinformatics Science and Technology, Harbin Medical University, Harbin, China; ^4^Epidemiology and Statistics, College of Public Health, Zhengzhou University, Zhengzhou, China; ^5^General Intensive Care Unit, The First Affiliated Hospital of Zhengzhou University, Zhengzhou, China

**Keywords:** sepsis-related neurodamage, metformin, gut microbiota, metabolites, CLP

## Abstract

Gut microbiota affects the functions of brains. However, its mechanism in sepsis remains unclear. This study evaluated the effect of metformin on ameliorating sepsis-related neurodamage by regulating gut microbiota and metabolites in septic rats. Cecal ligation and puncture (CLP) was used to establish the sepsis-related neurodamage animal models. Metformin therapy by gavage at 1 h after CLP administration was followed by fecal microbiota transplantation (FMT) to ensure the efficacy and safety of metformin on the sepsis-related neurodamage by regulating gut microbiota. The gut microbiota and metabolites were conducted by 16S rRNA sequencing and liquid chromatography-tandem mass spectrometry metabolomic analysis. The brain tissue inflammation response was analyzed by histopathology and reverse transcription-polymerase chain reaction (RT-PCR). This study reported brain inflammatory response, hemorrhage in sepsis-related neurodamage rats compared with the control group (C group). Surprisingly, the abundance of gut microbiota slightly increased in sepsis-related neurodamage rats than C group. The ratio of *Firmicutes/Bacteroidetes* was significantly increased in the CLP group than the C group. However, no difference was observed between the CLP and the metformin-treated rats (MET group). Interestingly, the abundance of *Escherichia_Shigella* increased in the MET group than the C and CLP groups, while *Lactobacillaceae* abundance decreased. Furthermore, *Prevotella_9*, *Muribaculaceae*, and *Alloprevotella* related to short-chain fatty acids production increased in the sepsis-related neurodamage of metformin-treated rats. Additionally, *Prevotella_9* and *Muribaculaceae* correlated positively to 29 metabolites that might affect the inflammatory factors in the brain. The FMT assay showed that metformin improved sepsis-related neurodamage by regulating the gut microbiota and metabolites in septic rats. The findings suggest that metformin improves the sepsis-related neurodamage through modulating the gut microbiota and metabolites in septic rats, which may be an effective therapy for patients with sepsis-related neurodamage.

## Introduction

Sepsis, characterized by systematic inflammation and abnormal immune response, leads to life-threatening organ dysfunction, with a high incidence and mortality rate (1). More than 1.5 million patients suffer from sepsis every year, resulting in about 33% mortality in the United States (2). Sepsis activates the host’s immune response, causing an inflammatory storm and subsequent abnormal immune response (3). Sepsis-associated encephalopathy (SAE) is a brain disease characterized by cognitive dysfunction, secondary to extrabrain infection, with an incidence rate of over 70% (4, 5). There are reports of neuroinflammation in SAE pathogenesis (6–8) and activated microglia during sepsis (9, 10). The brain endothelial cell activation and increased neutrophil infiltration could promote neuroinflammation in sepsis, inducing brain dysfunction (11). These results suggest that anti-inflammation therapy may be a potential method for attenuating SAE.

Gut microbiota plays a substantial role in cognitive function *via* the gut–brain axis. The abnormal gut microbiota changes are closely related to various brain diseases, especially cognitive impairment (12–14). Therefore, the regulatory effect of metformin on the gut microbiota might contribute to brain function improvement (15). Metformin is considered a first-line anti-diabetes agent, decreases insulin and insulin-associated factors, inhibits mitochondrial function, improves the metabolic and cellular processes (16), and cellular senescence (17). Orally administered metformin remains concentrated primarily in the gut (18), changing the main composition of gut microbiota (19). Transferring the feces of metformin-treated patients to germ-free mice ensures the efficacy of metformin on gut microbiota through altering the homeostasis of metalloproteins or metal transporters (20). Metformin increases short-chain fatty acid (SCFA) production in gut microbiota, enhances intestinal barrier function, and restores the gut microbial composition, thus improving the host metabolism (21, 22). This study investigated the regulatory effect of metformin on gut microbiota and its metabolites in relieving neuroinflammation in septic rats to provide an effective sepsis-related neuroinflammation method.

## Methods and Materials

### Animal Experiment

Forty-eight adult male Sprague-Dawley rats were purchased from Beijing Vital River Laboratory Animal Technology (Beijing, China). All rats were kept in a specific pathogen free (SPF) laboratory at 25 ± 2°C and in a 12/12 h light/dark cycle with free access to food and water. The adult male rats were randomly divided into 5 groups: sham-operated (C group, *n* = 10), cecal ligation and puncture induced sepsis-related neurodamage (CLP group, *n* = 10), metformin treatment (100 mg/kg, dissolved in purified water) through gavage 1 h after CLP administration (MET group, *n* = 10), the FMT-CLP group and FMT-MET group were pseudo-germ free rats with stools transplanted from the rats of CLP (*n* = 8) and MET (*n* = 10) groups for 5 days, inducing the sepsis-related neurodamage. The CLP-induced sepsis-related neurodamage models were based on the previous study (23). We injected pentobarbital (30 mg/kg) intraperitoneally to anesthetize the rats, ligated half of the cecum, and punctured the cecum twice using a 21-gauge needle. The cecum contents were squeezed out, repositioned, and the incision was closed using a two-layer suture (the muscle layer and skin). Furthermore, the rats were injected subcutaneously with saline (37°C; 1 mL/100 g) and rewarmed for 1 h before returning to the cage. The rats were fed with food and water and kept in a 12/12 h light/dark cycle. The C group rats had only an abdominal incision; the ceca were neither ligated nor punctured. All animal experiments were performed per the National Institutes of Health guidelines and with permission from the local Animal Care and Use Committee of Zhengzhou University (Henan, China).

### FMT Experiments

The FMT experiments were conducted as described in earlier papers (24–26). The antibiotics (vancomycin, 100 mg/kg; neomycin sulfate, 200 mg/kg; metronidazole, 200 mg/kg; and ampicillin, 200 mg/kg) were intragastrically injected into 16 adult male SD rats once a day for 5 days to deplete the gut microbiota, resulting in pseudo-germ free rats. We collected the donor rats’ (CLP and MET groups) feces and resuspended them in phosphate buffer saline (PBS) at 0.125 g/mL; 0.15 mL of this suspension was then given to rats by oral gavage once a day for 3 days. After 3 days, the tissues were collected at 24 h after CLP-induced sepsis.

### Histological Analysis

The hippocampus brain tissues were washed with sterile saline, fixed in 4% paraformaldehyde, and embedded in paraffin for hematoxylin and eosin (H&E) staining. The tissues were sectioned at 3–5 µm and stained with H&E for light microscopic analysis. Three sections and three regions of each section of the hippocampus brain tissues were quantified for edema and hemorrhage. Furthermore, the brain tissue paraffin sections were used in immunofluorescence analysis to evaluate the expression of iBA1 and GFAP. Briefly, the sections were boiled in citric acid buffer (Beyotime, Shanghai, China) for 20 min. After washing with PBS (Beyotime) three times, the brain sections were immersed in Triton X-100 (Beyotime) and subsequently blocked in 5% serum. Afterwards, they were incubated with primary antibodies at 4°C overnight. The brain sections were rinsed and coincubated with corresponding fluorescence-conjugated secondary antibodies in the dark, followed by staining with DAPI (Beyotime). Finally, the images of colon sections were captured under an epifluorescence microscope (Olympus, U-RFL-T, Japan).

### Reverse Transcription-Polymerase Chain Reaction Expression

TRIzol reagent (Takara, Tokyo, Japan) was used to extract the hippocampus from the brain tissue RNA following the manufacturer’s instructions. The TaqMan reverse transcription kit (UE, Suzhou, China) and a Gene Amp PCR system were used to generate cDNA. The PCR was performed with the RT-PCR superMIX (Yeasen, Shanghai, China). The GAPDH expression was used for gene expression normalization. The genes primers were: tumor necrosis factor-alpha (TNF-α) forward primer: CGTCAGCCGATTTGCCATTT, reverse primer: CTCCCTCAGGGGTGTCCTTA; IL-6 forward primer: AGAGACTTCCAGCCAGTTGC, reverse primer: AGTCTCCTCTCCGGACTTGT; CXCL1 forward primer: CGCTCGCTTCTCTGTGCA, reverse primer: TTCTGAACCATGGGGGCTTC; GAPDH forward primer: TGTGAACGGATTTGGCCGTA, reverse primer: GATGGTGATGGGTTTCCCGT.

### 16S rRNA Gene Sequencing

We used the cetyl trimethyl ammonium bromide/sodium dodecyl sulfate (CTAB/SDS) method and 1% agarose gels to extract and purify DNA, and sterile water to dilute the DNA concentration to 1 ng/μL. A specific primer (341F:CCTAYGGGRBGCASCAG; 806R:GGACTACNNGGGTATCTAAT) with the barcode was used to amplify the 16S rRNA genes of distinct regions (V3-V4). The PCR experiments were conducted using the Phusion^®^ High-Fidelity PCR Master Mix (New England Biolabs); the PCR samples were mixed with the same volume of 1X loading buffer and subjected to electrophoresis on 2% agarose gel for detection. Then, the Qiagen Gel Extraction kit (Qiagen, Germany) was used to purify the PCR product mixture. Additionally, the TruSeq^®^ DNA PCR-Free Sample Preparation kit (Illumina, USA) was used to generate the sequencing libraries following the manufacturer’s recommendations. The Qubit^®^ 2.0 Fluorometer (ThermoScientific) and Agilent Bioanalyzer 2100 system were used to evaluate the library quality. Finally, the Illumina NovaSeq 6000 platform was used to sequence the library and generate the 250 bp paired-end reads. The raw tags were double-ended reads, which could be accessed through fastq join (version: 1.3.1) (https://code.google.com/p/ea-utils/). A quality Phred score ≥ Q20 or ≥ Q30 was used to assess the DNA quality, and the USEARCH (version 11.0.667) (http://www.drive5.com/usearch/) program was used to cluster the ambiguous bases according to 97% similarity sequence. Additionally, Mothur v.1.42.1, the vegan package of R software and PICRUSt2 software package (https://github.com/picrust/picrust2) (27), was used to calculate the alpha and beta diversity and PICRUSt2.

### Ultra-High Performance Liquid Chromatography-Tandem Mass Spectrometry

Ultra-performance liquid chromatography-tandem mass spectrometry (UPLC-MS/MS) was used for fecal metabolomic profiling analysis. Fecal samples were prepared with the help of Q Exactive mass spectrometer (Thermo Scientific, MA, USA). A small amount (1/10 m/vol) of ice-cold 80% methanol (vol/vol) was added to each fecal product, vortexed for 10 min at room temperature, and incubated for 30 min at –20°C. Furthermore, the fecal samples were centrifuged at 14000*g*, 4°C for 15 min, and the supernatant was transferred to a fresh microcentrifuge tube. The QC sample preparation method involved extracting the same volume of supernatant from each sample, mixing and centrifuging, and then transferring it to a mass spectrometry bottle. The LC-MS/MS analysis was conducted in the data correlation acquisition (DCA) mode using vanquish UHPLC system (Thermo Fisher) and Orbitrap Q active hf-x mass spectrometer (Thermo Fisher). The sample was injected into an Accucore HILIC column (100 mL) at a flow rate of 0.3 mL/min in a linear gradient of 20 min × 2.1 mm (2.6 µm). The eluents in the positive polarity mode were eluent A (0.1% FA, 10 mM ammonium acetate in 95% ACN) and eluent B (0.1% FA, 10 mM ammonium acetate in 95% ACN). The solvent gradient was set as follows: 2% B, 1 min; 2–50% B, 16.5 minutes; 50–2% boron, 2.5 min. The Q-Exactive HF-X mass spectrometer operated under the positive and negative polarity modes, the spray voltage was 3.2 kV, the capillary temperature was 320°C, the sheath gas flow rate was 35 Arb, and the auxiliary gas flow rate was 10 Arb. Compound Discover v3.1 (CD) software was used for data extraction and processing. Compound identification included online search through mzcloud and chemspider, in addition to the built-in search database of the software with an endogenous metabolite database of 4400 compounds. It included the high-resolution mass spectrometry database Orbitrap traditional Chinese medicine library (otcml) of traditional Chinese medicine components in mzVault library. Specific metabolites were identified through differential metabolite screening, multivariate statistical analysis (OPLS-DS, threshold *P*<0.05, VIP>1), and univariate statistical analysis (*t*-test, only *P*<0.05 screening). If specific metabolites were discussed with the help of path information, the Kyoto Encyclopedia of Genes and Genomes (KEGG) path screening was added. The constant sum algorithm was used to realize sample standardization. After log2 logarithmic conversion, we used the automatic scaling algorithm to complete normalization.

### Statistical Analyses

The R-package (https://www.r-project.org/) and GraphPad Prism version 5.0 (GraphPad Software, La Jolla, CA, USA) software were used for statistical analyses. Mean ± standard deviation was used to assess the quantitative data, and the variance was used to analyze the comparison among more than three groups (C vs CLP vs MET vs FMT-CLP vs FMT-MET). We used the *t*-test or two-way analysis of variance (ANOVA) and Bonferroni post-test instead of individual comparisons to statistically analyze the data. Alpha diversity was calculated using Mothur v1.42.1, beta diversity was calculated using permutational ANOVA to compare groups (vegan package in R-package), and the pathway enrichment was calculated using PICRUSt2 software package (https://github.com/picrust/picrust2). Furthermore, the Spearman’s rank correlation coefficients were calculated for correlation analysis, and the R-package Corrplot, ggcorplot, and pheatmap were used for the correlation matrix visualization. All results among the groups were analyzed with a statistical significance level set at *P*<0.05.

## Results

### Metformin Mitigates Sepsis-Related Neuroinflammation and Injury in Septic Rats

**Figure 1:**
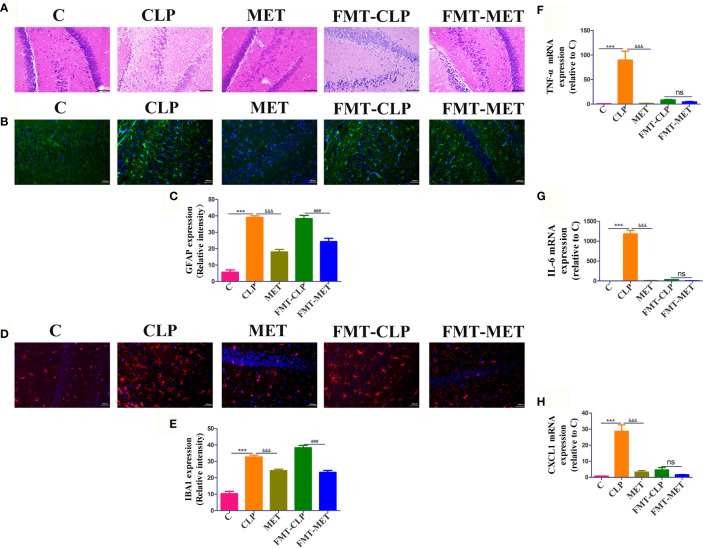
Metformin mitigates sepsis-related neuroinflammation and injury in septic rats. **(A)** H&E staining showed a large number of nerve cells with nuclear pyknosis, disordered structural arrangement, edema of nerve cells, and serious injury in CLP group compared with the C group; metformin administration reversed the CLP-induced serious brain injury compared to the septic rats. It showed a decreased level of the inflammatory infiltrates, oedema, and haemorrhage of brain tissue in the FMT-MET group compared to the FMT-CLP group (scale bar = 100 um). **(B–E)** The immunofluorescence experiments indicated that the iBA1 and GFAP expression increased significantly in septic rats compared to the C group rats, whereas metformin administration and metformin-treated FMT assay showed significantly decreased iBA1 and GFAP expression (scale bar = 100 um). **(F–H)** The expressions of TNF-α, IL-6, and CXCL1 increased drastically in the CLP group than the MET group. * CLP vs C group; & MET vs CLP group; : FMT-MET vs FMT-CLP group, ***, ###, &&& *P*<0.001; ns: not significant (*n* = 3–5/group).

The histological and RT-RNA analyses of brain tissues were conducted 24 h post-CLP to detect neuroinflammation and injury in CLP-induced septic rats. The results showed a large number of nerve cells with nuclear pyknosis, disordered structural arrangement, edema of nerve cells, and serious injury in CLP-induced septic rats compared with the C group; metformin administration reversed the CLP-induced nerve cells with nuclear pyknosis, disordered structural arrangement, edema of nerve cells, and serious brain injury compared to the septic rats. Furthermore, the results of FMT-CLP and FMT-MET groups were similar to those of CLP and MET groups (**Figure 1A**). We tested the effect of metformin on the expression of neuro microglia of brain tissue in septic rats. The immunofluorescence experiments indicated that the iBA1 and GFAP expression increased significantly in septic rats compared to the C group rats, whereas metformin administration and metformin-treated FMT assay showed substantially decreased iBA1 and GFAP expression (*P*<0.05; **Figures 1B–E**). Furthermore, we checked the mRNA expressions of TNF-α, chemokine [C-C motif] ligand 1 (CXCL1), interleukin-6 (IL-6) of the septic rats’ brain tissue to assess the effect of metformin on the inflammatory response of brain tissue. The results showed that the expression of TNF-α, IL-6, and CXCL1 increased drastically in the CLP group than the MET group. These inflammatory factors in the FMT-CLP group increased compared to the FMT-MET group, but there were no significance (*P>*0.05; **Figures 1F–H**). Together, these results revealed that metformin could relieve neuroinflammation and injury in septic rats.

### Effect of Metformin on Gut Microbiota in Septic Rats

**Figure 2:**
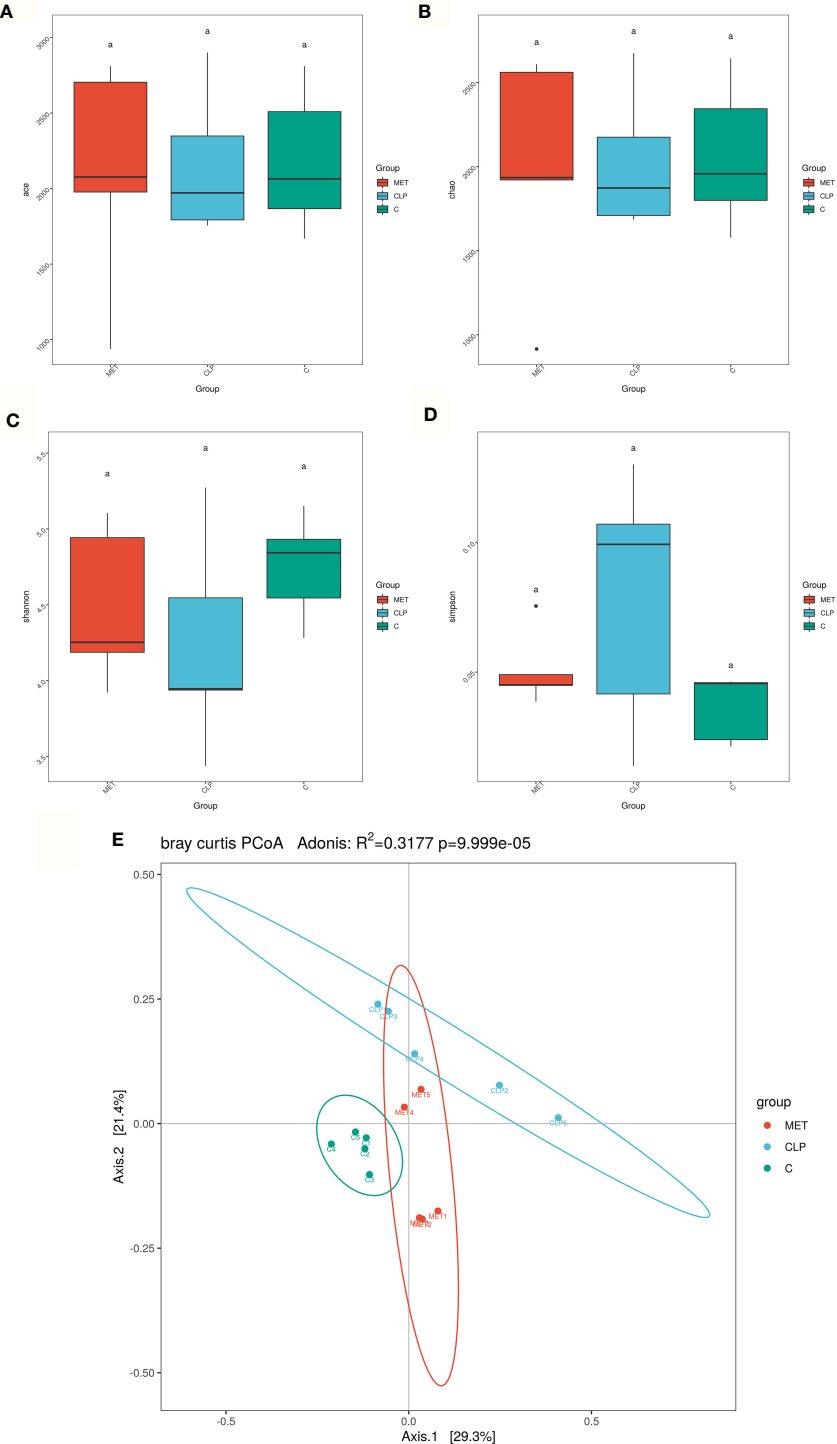
Effect of metformin on gut microbiota in septic rats. **(A–D)** 16S rRNA analysis showed that the alpha diversity of gut microbiota such as Ace, Chao1, Shannon and Simpson index was slightly reduced in the CLP group compared to the C group. However, metformin treatment slightly reversed the alpha diversity reduction caused by sepsis. **(E)** The beta diversity of gut microbiota showed that the compositions in the CLP group were different from the C group. However, the composition of the gut microbiota in the MET group was similar to the C group. a P<0.05 (*n* = 5/group).

**Figure 3:**
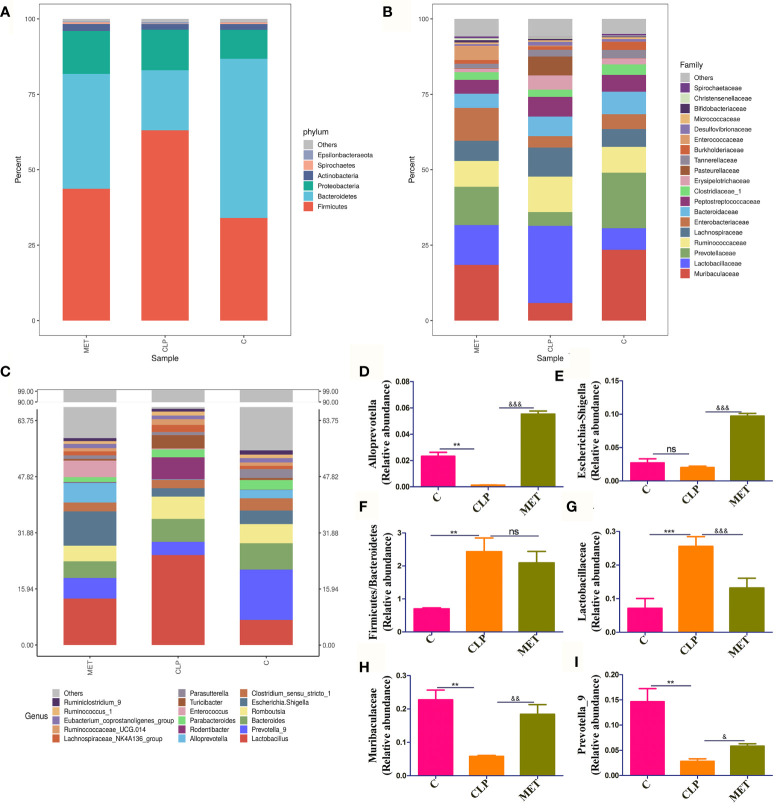
The beneficial effect of metformin on gut microbiota in septic rats. **(A–C)** The gut microbiota at Phylum, Family, and Genus levels showed the difference among the C, CLP, and MET groups. **(D–I)** The specific gut microbiota abundance varied among the groups, such as the relative abundance of *Alloprevotella*, *Escherichia_Shigella*, *Prevotella*_9 and *Muribaculaceae* were decreased in the CLP group compared to the C group, but increased in the MET group compared to the CLP group rats; while ratio of *Firmicutes*/*Bacteroidetes* and *Lactobacillus* were increased in the CLP group compared to the C group, and decreased in the MET group.*: CLP vs C group; & MET vs CLP group. & P<0.05; **, && P<0.01; *** , &&& P<0.001; ns, not significant (*n* = 5/group).

Gut analysis was performed to assess the composition of gut microbiota and the impact of metformin on the gut microbiota of septic rats. Our results revealed that the alpha diversity of gut microbiota (Ace, Chao, Shannon) was slightly reduced in the septic rats compared to the C group. However, metformin treatment slightly reversed the alpha-diversity reduction caused by sepsis (**Figures 2A–D**), suggesting that metformin increased the alpha diversity of gut microbiota induced by sepsis. Additionally, we tested the gut microbiota beta diversity of all rats. The results showed that the gut microbial composition of metformin-treated septic rats was more similar to the C group rats than the septic rats (**Figure 2E**). Comparatively, the *Firmicutes/Bacteroidetes* ratio and the relative abundance of *Lactobacillaceae* increased in the CLP group compared to the C group. However, metformin administration slightly reduced the *Firmicutes/Bacteroidetes* ratio and the relative abundance of *Lactobacillaceae* compared to the CLP group (**Figures 3A, F**). Surprisingly, the relative abundance of *Escherichia_Shigella*, related to lipopolysaccharide (LPS) production, in the CLP group increased with metformin administration (**Figures 3C, E**). The relative abundance of *Alloprevotella, Muribaculaceae* and *Prevotella_9*, related to SCFA production and anti-inflammation, decreased in the CLP group but increased in metformin-treated septic rats compared to the CLP group rats, while the relative abundance of Lactobacillaceae increased in CLP group compared with C and MET group. (*P*<0.05; **Figures 3B–D, G–I**). Collectively, these findings highlight the modulatory effect of metformin on the gut microbiota of septic rats.

**Figure 4:**
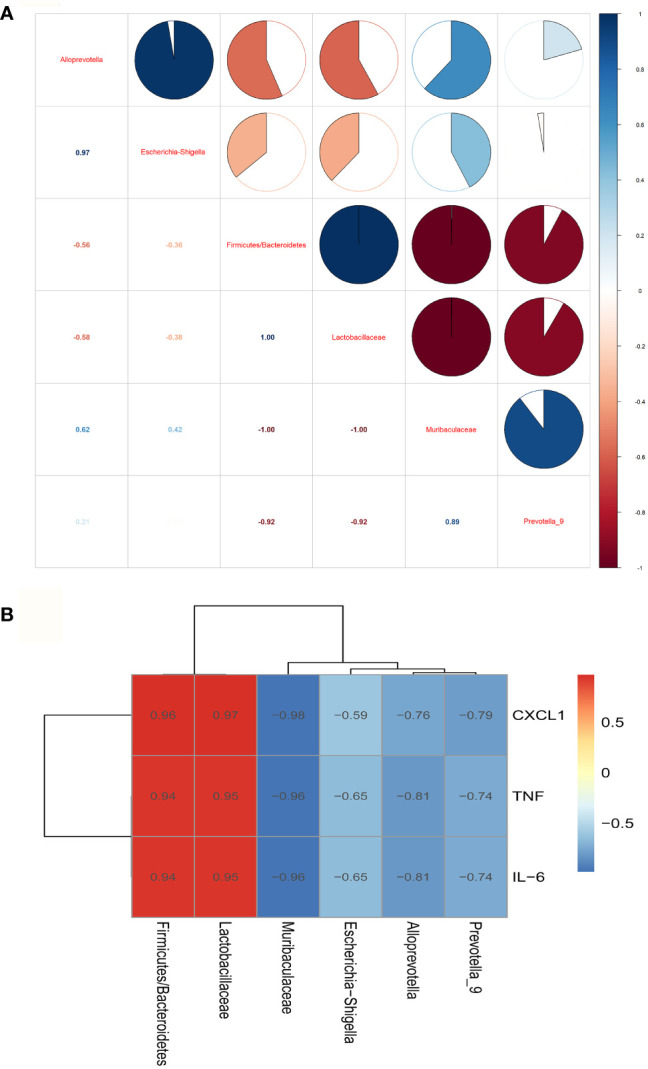
The correlation analysis of the gut microbiota and inflammation of the brain of septic rats. **(A)** The correlation analysis of the gut microbiota showed that *Muribaculaceae* and *Alloprevot_9* were negatively correlated to *Firmicutes/Bacteroidetes* and *Lactobacillaceae*, and *Muribaculaceae* was positively correlated to *Alloprevot_9*.Interestingly, *Escherichia_Shigella* showed a positive correlation to *Alloprevotella.* **(B)** The correlation analysis between gut microbiota and brain inflammation showed that *Firmicutes/Bacteroidetes* and *Lactobacillaceae* were positively and *Alloprevotella*, *Muribaculaceae*, Prevotella_9, and *Escherichia_Shigella* were negatively correlated to TNF, IL-6, and CXCL1. TNF:TNF-α.

The correlation analysis results among the groups about gut microbiota showed that *Muribaculaceae* and *Alloprevot_9* were negatively correlated to *Firmicutes/Bacteroidetes* and *Lactobacillaceae*, and *Muribaculaceae* was positively correlated to *Alloprevot_9*. Interestingly, *Escherichia_Shigella* showed a positive correlation to *Alloprevotella* (**Figure 4A**). We further explored the correlation between gut microbiota and inflammation and observed that *Firmicutes/Bacteroidetes* and *Lactobacillaceae* were positively and *Alloprevotella, Muribaculaceae*, *Prevotella_9*, and *Escherichia_Shigella* were negatively correlated to TNF-α, IL-6, and CXCL1 (**Figure 4B**). The PICRUSt analysis used to evaluate the differences in the COG_metagenome among the groups showed that CLP-induced sepsis enriched 8 COG_metagenomes negatively, while 30 COG_metagenomes showed a positive enrichment compared with the C group. However, metformin influenced 29 COG_metagenome enrichment compared to the CLP group (**Supplementary Figure 1**). Therefore, it can be inferred that metformin regulated gut microbiota and affected the metabolic pathways in septic rats.

### Metformin Affects the Gut Microbial Metabolites in Septic Rats

**Figure 5:**
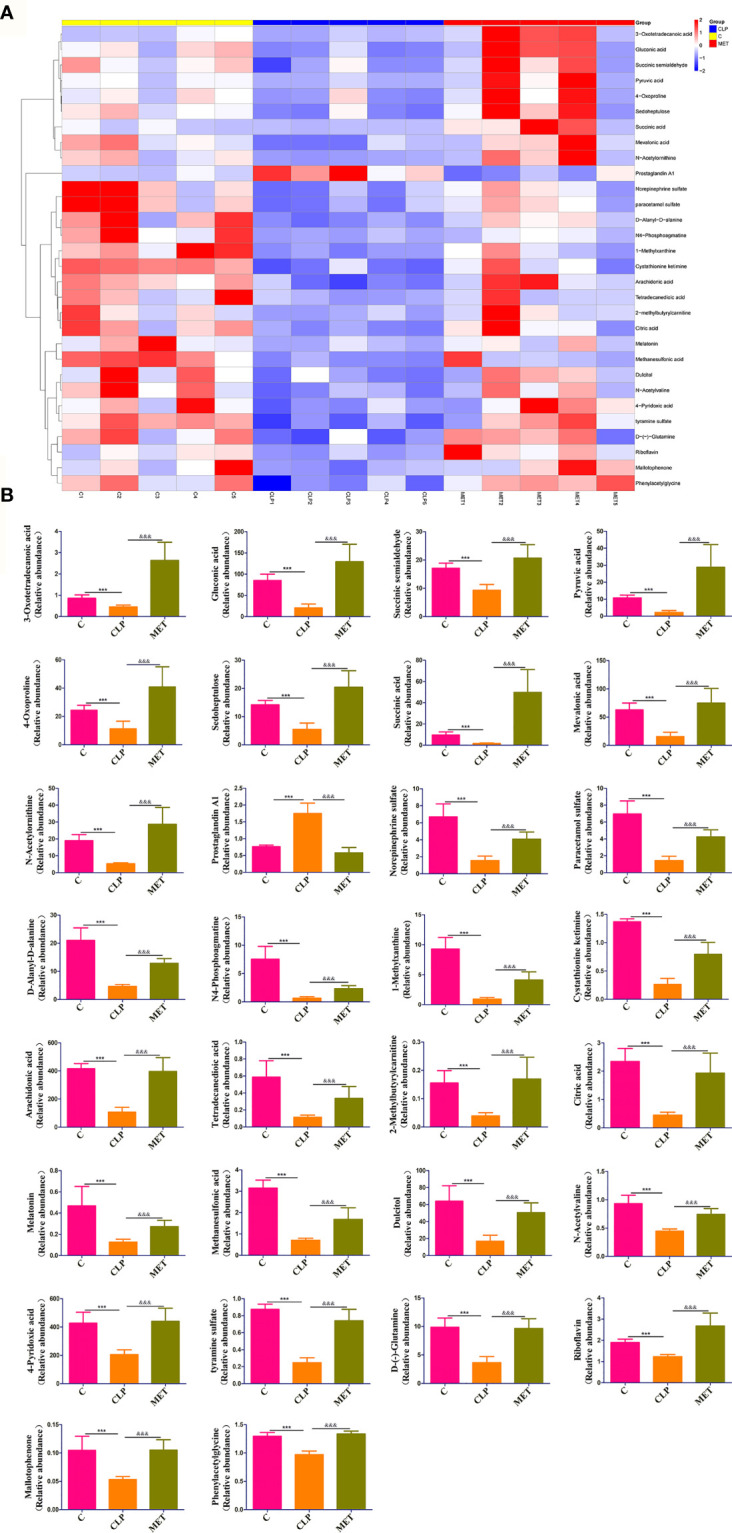
Metformin affects the gut microbial metabolites in septic rats. **(A)** Untargeted metabolic analysis showed differences in the intensity of specific metabolites among the groups. **(B)** 30 specific metabolites showed significant differences among the groups. *: CLP vs C group; & MET vs CLP group. ***, &&& P<0.001 (*n* = 5/group).

**Figure 6:**
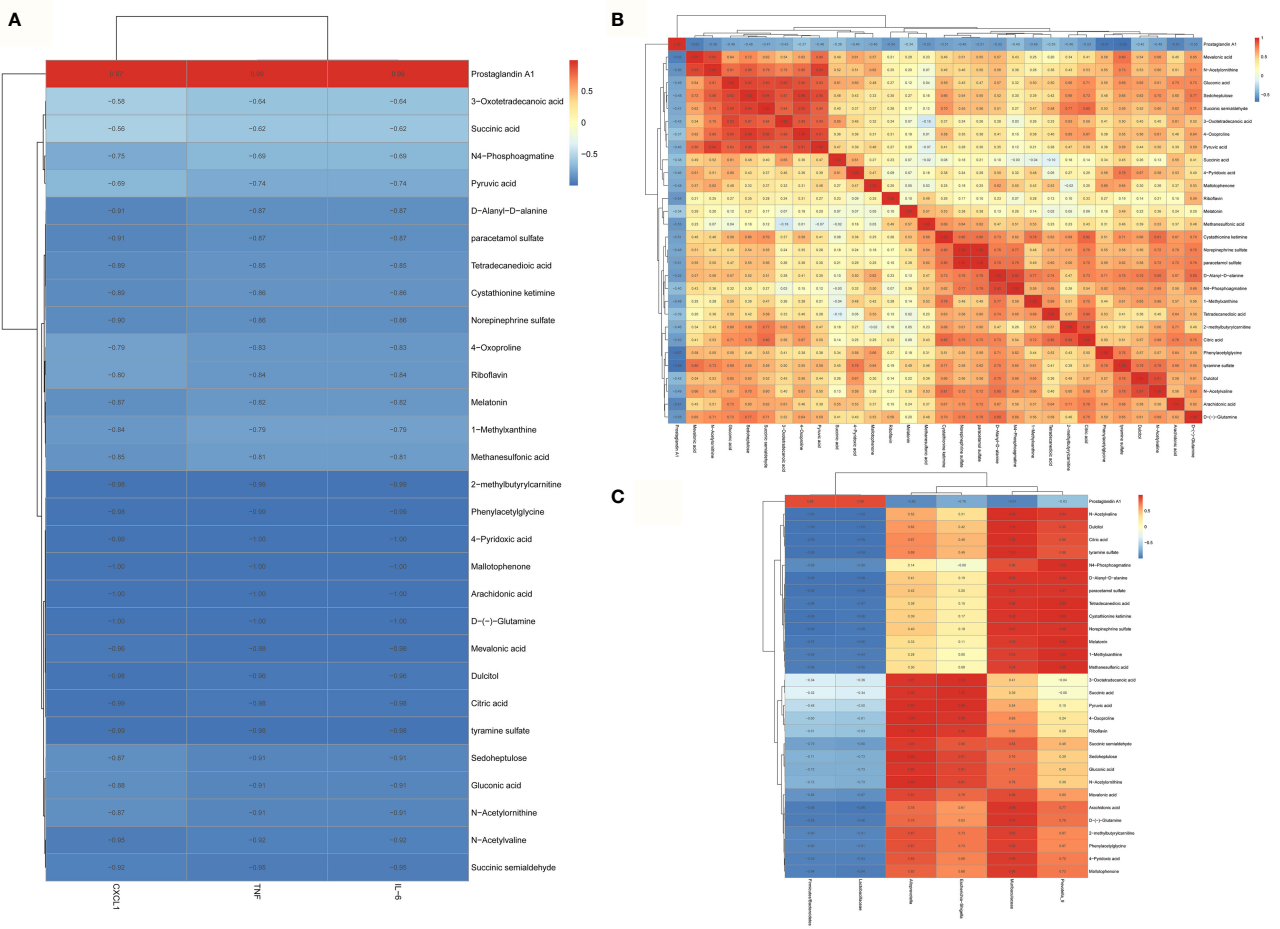
The correlation analysis of the gut microbial metabolites, gut microbiota and brain inflammation in septic rats. **(A)** A correlation analysis showed 21 positive and 29 negative correlations among the microbial metabolites. **(B)** only prostaglandin A1 correlated positively with the inflammatory factors and chemokines such as TNF, IL-6, and CXCL1 of the brain. TNF:TNF-α **(C)** The correlation analysis between gut microbiota and its metabolites showed a positive correlation of *Firmicutes*/*Bacteroidetes*, whereas *Lactobacillaceae* showed a positive correlation with prostaglandin A1 and a negative correlation with the remaining 29 metabolites.

Metabolites of gut microbiota are functional units that affect host cells and are produced during food fermentation. Untargeted metabolomics of the fecal samples was performed to explore the regulation of gut microbial metabolites by metformin. The fecal metabolites were affected by sepsis, whereas metformin administration induced distinct metabolite profile clusters in septic rats (**Supplementary Figure 2**). We further explored the impact of metformin on microbial metabolites of septic rats. The results showed a significant increase in prostaglandin A1 levels, while the abundant 1-methylxanthine, 2-methylbutyrylcarnitine, 3-oxotetradecanoic acid, 4-oxoproline, 4-pyridoxic acid, arachidonic acid, citric acid, cystathionine ketimine, D-(-)-glutamine, D-alanyl-D-alanine, dulcitol, gluconic acid, mallotophenone, melatonin, methanesulfonic acid, mevalonic acid, N4-phosphoagmatine, N-acetylornithine, N-acetylvaline, norepinephrine sulfate, paracetamol sulfate, phenylacetylglycine, pyruvic acid, riboflavin, sedoheptulose, succinic acid, succinic semialdehyde, tetradecanedioic acid, tyramine sulfate decreased in the CLP group compared to the C group. However, metformin could reverse the sepsis-induced changes in the metabolites mentioned above (*P*<0.05; **Figures 5A, B**). A correlation analysis showed 21 positive and 29 negative correlations among the microbial metabolites (*P*<0.05; **Figure 6A**). The correlation analysis between gut microbiota and its metabolites showed a positive correlation of *Firmicutes/Bacteroidetes*, whereas *Lactobacillaceae* showed a positive correlation with prostaglandin A1 and a negative correlation with the remaining 29 metabolites (*P*<0.05; **Figure 6B**). Furthermore, *Alloprevotella*, and *Escherichia_Shigella* correlated positively with 2-methylbutyrylcarnitine, 3-oxotetradecanoic acid, 4-oxoproline, 4-pyridoxic acid, arachidonic acid, D-(-)-Glutamine, gluconic acid, mallotophenone, mevalonic acid, N-acetylvaline, phenylacetylglycine, pyruvic acid, riboflavin, sedoheptulose, succinic acid, succinic semialdehyde; while *Muribaculaceae* and *Alloprevot_9* correlated positively with 1-methylxanthine, citric acid, cystathionine ketimine, D-alanyl-D-alanine, dulcitol, melatonin, methanesulfonic acid, N4-phosphoagmatine, N-acetylornithine, norepinephrine sulfate, paracetamol sulfate, tetradecanedioic acid, tyramine sulfate (*P*<0.05; **Figure 6B**). Additionally, only prostaglandin A1 correlated positively with inflammatory factors and chemokines such as TNF-α, IL-6, and CXCL1 of the brain (*P*<0.05; **Figure 6C**). More importantly, the changed metabolites impacted the metabolic pathways of tyrosine, metabolic pathways, biosynthesis of various secondary metabolites, and so forth (**Supplementary Figure 3****)**. Collectively, the findings highlight the role of metformin in regulating the sepsis-induced imbalance of gut microbiota and its metabolites.

## Discussion

This study revealed that metformin could mitigate sepsis-related neurodamage by regulating gut microbiota and its metabolites in septic rats. The composition of gut microbiota and metabolites in septic rats treated with metformin increased the abundance of gut microbiota related to the SCFA production and anti-inflammation. Our work supported the general idea of using FMT analysis in sepsis, as metformin protects inflammatory response and neurodamage in sepsis.

Metformin is the first-line treatment for type 2 diabetes, and its other effects have been widely studied. Metformin has attracted extensive attention for its impact on intestinal microbiota in various diseases. A study reported that metformin could delay the aging of nematodes by changing the metabolism of microbial folate and methionine, providing effective treatment for aging (28). A subsequent study showed that metformin played an anti-diabetic role by regulating the encoding of metal protein or metal transporter encoded by species (20). A recent article reported that metformin could regulate lipid metabolism and affect the host’s life span (27). More and more evidence shows that the microbiome changes after metformin administration have been previously described and related to some host effects of the drug; metformin affects sepsis at least in part by regulating the microbiome.

Increased abundance of *Escherichia coli* (*E. coli*) after metformin treatment was observed in healthy volunteers and patients with type2 diabetes mellitus (T2DM) or other diseases (20, 29–32). Additionally, a study reported that metformin administration uses other energy sources to create a competitive environment for *E. coli*, resulting in many gut microbial changes (30, 33). A subsequent study pointed out that metformin administration may affect the relative abundance of *E. coli* under *in vitro* intestinal simulation conditions (20). Another recent article demonstrated that the large presence of *E. coli/Shigella* before metformin treatment was related to side effects (31). Furthermore, a report showed that *E. coli* was considered a marker of gastrointestinal side effects after metformin administration (30).

The FMT analysis emphasizes the role of gut microbiota as upstream conductors of sepsis-related neurodamage. We observed that metformin therapy relieved sepsis-related neurodamage by regulating gut microbiota and metabolites and alleviated gut leakage in septic rats, which was consistent with the previous study (15). It was reported earlier that gut leakage could regulate inflammation, a high-risk metabolic dysfunction in the elderly (34). The results were consistent with a previous article reporting that decreased expression of gut barrier markers such as tight junction proteins resulted in intestinal leakage (35). Additionally, CLP-induced colitis infiltration, edema, and bleeding also promoted intestinal leakage.

Our study suggested that there were significant differences in gut microbial composition between the C and CLP groups. As expected, metformin administration increased the abundance of *Escherichia_Shigella*. The increased levels of opportunistic pathogens with the proinflammatory response (such as *Klebsiella* and *Escherichia_Shigella* in CLP-induced sepsis) destroies the gut microecological balance, while the activated gut pathogens increase inflammation, which is consistent with the previous study (23). Another study reported that *Escherichia_Shigella* was either associated with obesity-related metabolic dysfunction or a proinflammatory bacterium (36). Our study also showed that *Escherichia_Shigella* and sepsis-related neural inflammation were positively correlated. The gut microbial composition of the MET group was similar to that of the C group. This study showed that compared to the CLP group, metformin therapy increased the abundance of *Muribaculaceae*, and *Alloprevotella* and *Prevotella_9*. It can produce energy providing SCFA for intestinal cells and maintain the intestinal barrier (37), thereby preventing the displacement of LPS from the intestinal barrier (38, 39). *Prevotella_9* and *Muribaculaceae* have beneficial effects on intestinal disorders by immune regulation and intestinal homeostasis regulation (21, 40). Metformin administration also affected COG_metagenomes by changing the intestinal microbiota, demonstrating the role of metformin in improving intestinal microbial imbalance by affecting metabolic pathways of tyrosine, biosynthesis of various secondary metabolites, and so forth.

Additionally, intestinal microbial metabolites are a response to changes in the intestinal microbiota. In our study, the intestinal microbial composition was similar between the groups because all rats were locked together before CLP. Therefore, the changes in intestinal microbiota are caused by sepsis. Different microbial components may not be parallel to the corresponding metabolic spectrum in sepsis (41). Among 30 differential metabolites, only one metabolite, prostaglandin A1 and neural inflammation showed a positive correlation; *Firmicutes/Bacteroidetes* and *Lactobacillaceae* showed a positive correlation with prostaglandin A1 while they were negatively correlated with the remaining 29 metabolites. Furthermore, *Alloprevotella* and *Escherichia_Shigella* correlated positively with 2-methylbutyrylcarnitine, 3-oxotetradecanoic acid, 4-oxoproline, 4-pyridoxic acid, arachidonic acid, D-(-)-glutamine, gluconic acid, mallotophenone, mevalonic acid, N-acetylvaline, phenylacetylglycine, pyruvic acid, riboflavin, sedoheptulose, succinic acid, and succinic semialdehyde; while *Muribaculaceae* and *Alloprevot_9* correlated positively with 1-methylxanthine, citric acid, cystathionine ketimine, D-alanyl-D-alanine, dulcitol, melatonin, methanesulfonic acid, N4-phosphoagmatine, N-acetylornithine, norepinephrine sulfate, paracetamol sulfate, tetradecanedioic acid, and tyramine sulfate. Thus, the metabolites changed in our study confirmed that metformin effectively inhibited intestinal microbial metabolic abnormalities caused by CLP.

The study has some shortcomings. First, the samples in each group are very small, and the results of this study need to be confirmed in further study. Furthermore, the metformin concentration in the feces of donors is not detected before the FMT assay. It may affect the result reliability as the unprocessed metformin in the fece samples may act directly on the recipient animals. Note that we do not confirm the host metabolites, so it may weaken the causality of metformin in improving neurodamage through gut microbiota and its metabolites. Therefore, further study should continue to explore the mechanism by which metformin ameliorates neurodamage through gut microbiota. Finally, we only perform untargeted metabolomics, which can provide a future scope for targeted metabolomics to confirm the specific metabolites that influence the pathophysiology in sepsis.

## Conclusion

Our results provided some evidence that metformin may alleviate neuroinflammation and injury *via* the restored gut microbiota and its metabolite dysbiosis. *Firmicutes/Bacteroidetes* and *Lactobacillaceae* and their related metabolite, prostaglandin A1, increased significantly in septic rats; metformin therapy could restore the gut microbiota dysbiosis and increase the relative abundance of *Muribaculaceae*, *Prevotella_9*, and *Alloprevotella* and their related metabolites. These results demonstrate that metformin could be a potential therapy for neuroinflammation and injury caused by sepsis-induced gut microbiota and metabolite dysbiosis in septic rats.

## Data Availability Statement

The datasets presented in this study can be found in online repositories. The name of the repository and accession number can be found below: NCBI BioProject; 815493.

## Ethics Statement

The animal study was reviewed and approved by the local Animal Care and Use Committee of Zhengzhou University.

## Author Contributions

HYZ and YJL were responsible for the design and preparation of the study, and drafted the manuscript. XFD was in charge of supervision of the experiments, and revising the manuscript draft. RQZ and GYS conducted the experiments, analyzed the data, and calculated the figures. All authors contributed to the article and approved the submitted version.

## Funding

This study was supported by the 2021 youth talent promotion project in Henan Province (Grant No. 2021HYTP053), the 2021 joint construction project of Henan Medical Science and Technology Breakthrough Plan (Grant No. LHGJ20210299).

## Conflict of Interest

The authors declare that the research was conducted in the absence of any commercial or financial relationships that could be construed as a potential conflict of interest.

## Publisher’s Note

All claims expressed in this article are solely those of the authors and do not necessarily represent those of their affiliated organizations, or those of the publisher, the editors and the reviewers. Any product that may be evaluated in this article, or claim that may be made by its manufacturer, is not guaranteed or endorsed by the publisher.

## Supplementary Material

Supplementary Figure 1 The PICRUSt analysis used to evaluate the differences in the COG_metagenome among the groups showed that CLP-induced sepsis enriched 8 COG_metagenomes negatively, while 30 COG_metagenomes showed a positive enrichment compared with the C group **(A)**. However, metformin influenced 29 COG_metagenome enrichment compared to the CLP group **(B)**.

Supplementary Figure 2 The metabolite analysis of Orthogonal Partial Least Squares Discrimination Analysis (OPLS-DA) and Permutation test to assess the difference of metabolites and whether the model was credible among the groups. **(A, B)** the OPLS-DA and permutation test in C group and CLP group. **(C, D)** the OPLS-DA and permutation test in CLP group and MET group.

Supplementary Figure 3 The untargeted whole metabolic analysis showed markedly different metabolite profiles in the C group, CLP group, and MET group. **(A)** showed the top metbolites profiles enriched pathways in C and CLP group. **(B)** showed the top metbolites profiles enriched pathways in MET and CLP group. The metabolite profiles in MET and C groups were similar compared with the CLP group.

## Abbreviations

SCFA, short chain fatty acid; LC-MS/MS, liquid chromatography-tandem mass spectrometry; FMT, fecal microbiota transplantation; CLP, cecal ligation and puncture; TNF-α, tumor necrosis factor-alpha; CXCL, chemokine[C-C motif] ligand; IL, interleukin; H&E, hematoxylin and eosin; PICRUSt, Phylogenetic Investigation of Communities by Reconstruction of Unobserved States.
