# Supplementary figures and images for: Metformin Mitigates Sepsis-Related Neuroinflammation via Modulating Gut Microbiota and Metabolites
Source: Front Immunol. 2022 Apr 29;13:797312. doi: 10.3389/fimmu.2022.797312 (PMC9102391; doi:10.3389/fimmu.2022.797312)

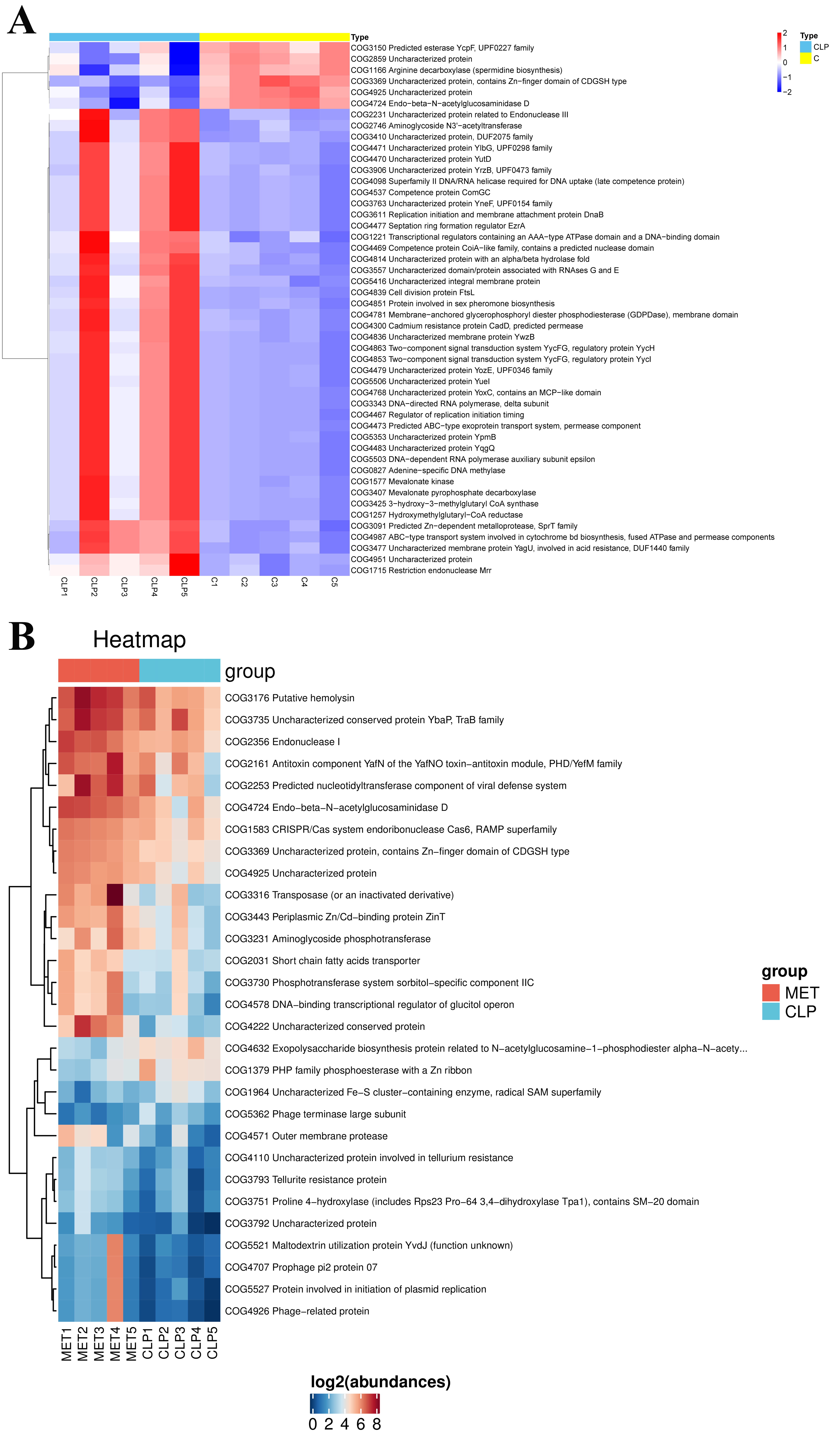

Supplement: Supplementary Figure 1 — The PICRUSt analysis used to evaluate the differences in the COG_metagenome among the groups showed that CLP-induced sepsis enriched 8 COG_metagenomes negatively, while 30 COG_metagenomes showed a positive enrichment compared with the C group (A). However, metformin influenced 29 COG_metagenome enrichment compared to the CLP group (B). [file Image_1.tif]

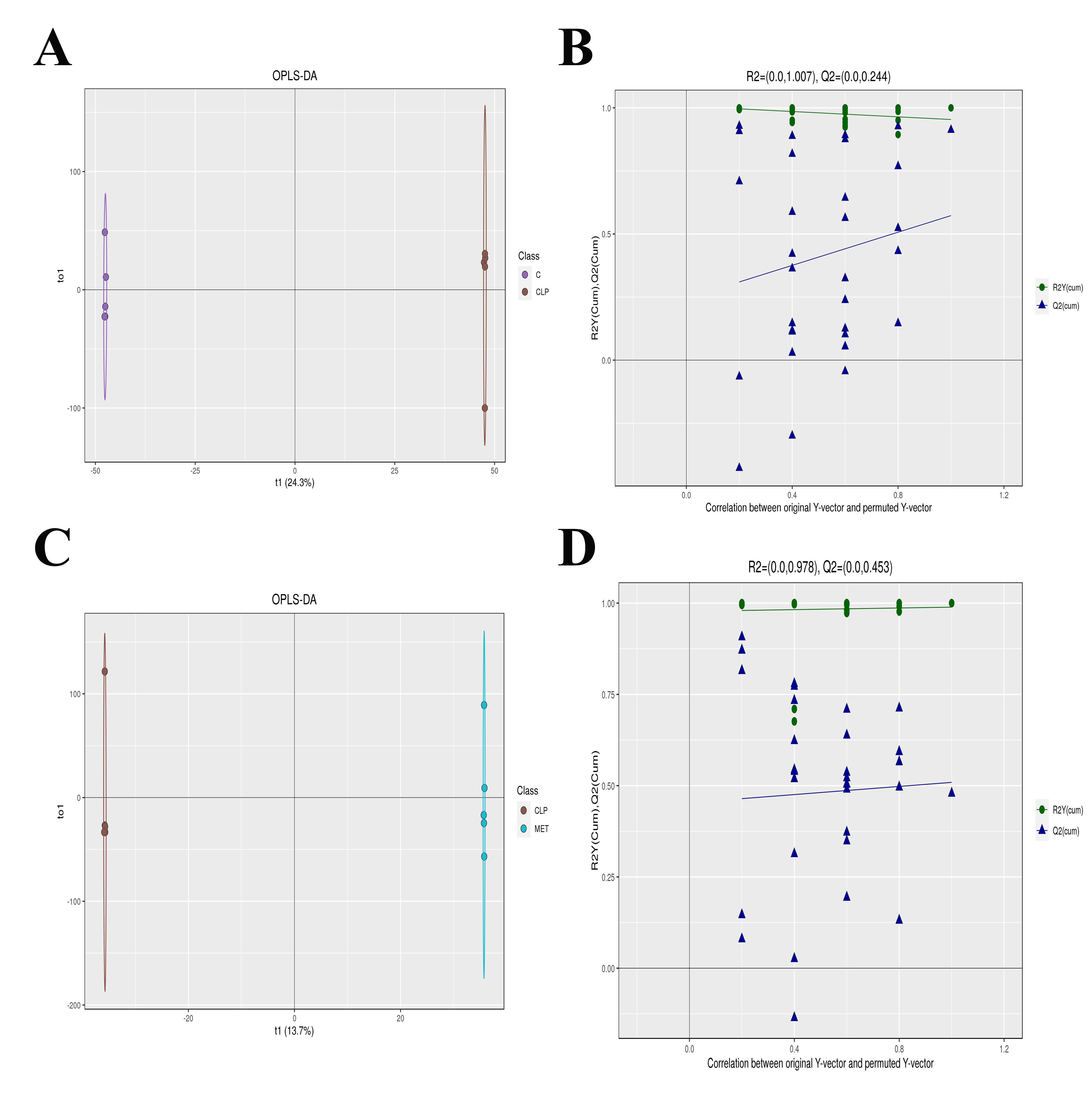

Supplement: Supplementary Figure 2 — The metabolite analysis of Orthogonal Partial Least Squares Discrimination Analysis (OPLS-DA) and Permutation test to assess the difference of metabolites and whether the model was credible among the groups. (A, B) the OPLS-DA and permutation test in C group and CLP group. (C, D) the OPLS-DA and permutation test in CLP group and MET group. [file Image_2.tif]

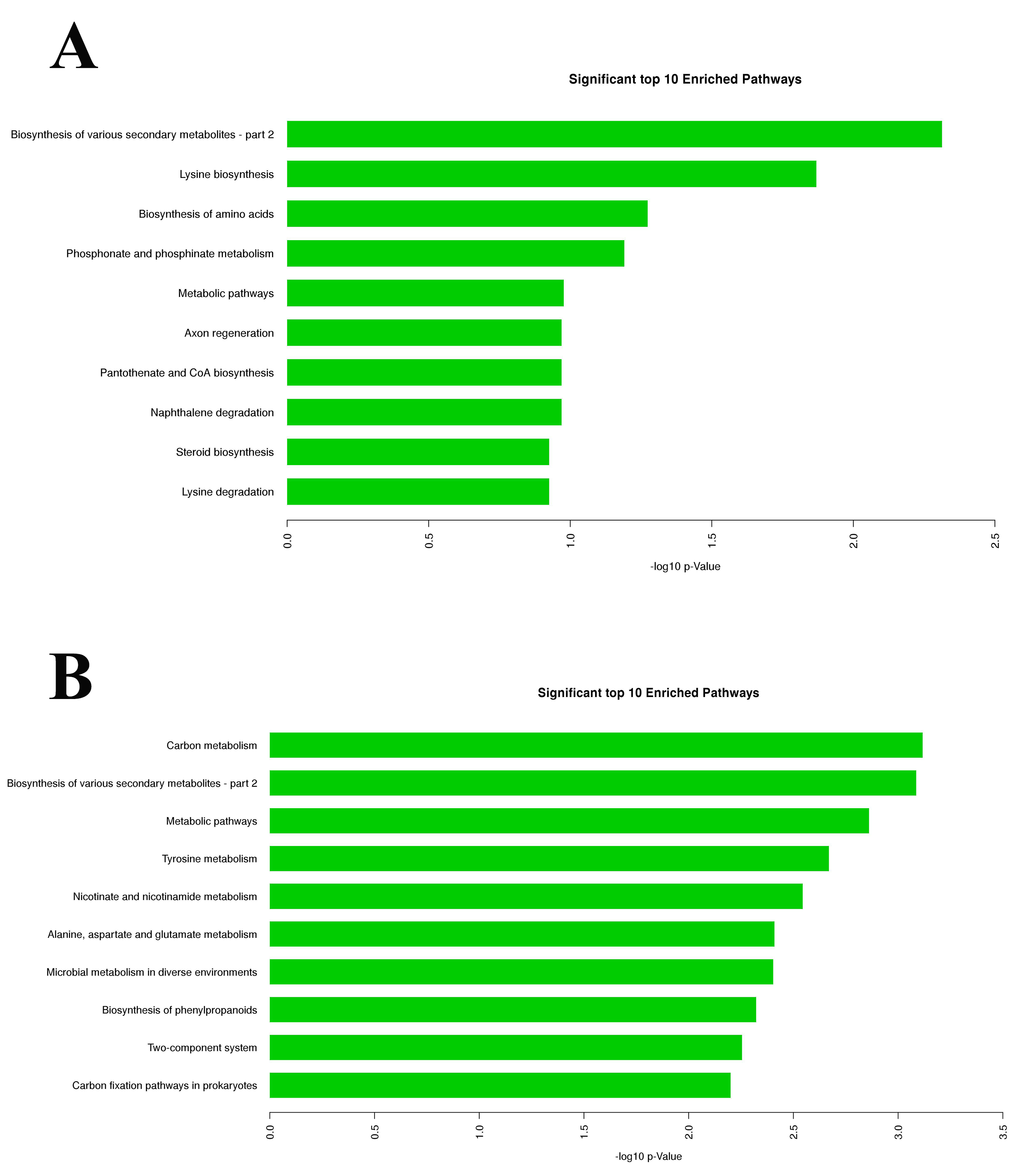

Supplement: Supplementary Figure 3 — The untargeted whole metabolic analysis showed markedly different metabolite profiles in the C group, CLP group, and MET group. (A) showed the top metbolites profiles enriched pathways in C and CLP group. (B) showed the top metbolites profiles enriched pathways in MET and CLP group. The metabolite profiles in MET and C groups were similar compared with the CLP group. [file Image_3.tif]
